# Foxn1 Transcription Factor Regulates Wound Healing of Skin through Promoting Epithelial-Mesenchymal Transition

**DOI:** 10.1371/journal.pone.0150635

**Published:** 2016-03-03

**Authors:** Barbara Gawronska-Kozak, Anna Grabowska, Anna Kur-Piotrowska, Marta Kopcewicz

**Affiliations:** Institute of Animal Reproduction and Food Research of Polish Academy of Sciences, Olsztyn, Poland; University of Alabama at Birmingham, UNITED STATES

## Abstract

Transcription factors are key molecules that finely tune gene expression in response to injury. We focused on the role of a transcription factor, Foxn1, whose expression is limited to the skin and thymus epithelium. Our previous studies showed that Foxn1 inactivity in nude mice creates a pro-regenerative environment during skin wound healing. To explore the mechanistic role of Foxn1 in the skin wound healing process, we analyzed post-injured skin tissues from Foxn1::Egfp transgenic and C57BL/6 mice with Western Blotting, qRT-PCR, immunofluorescence and flow cytometric assays. Foxn1 expression in non-injured skin localized to the epidermis and hair follicles. Post-injured skin tissues showed an intense Foxn1-eGFP signal at the wound margin and in leading epithelial tongue, where it co-localized with keratin 16, a marker of activated keratinocytes. This data support the concept that suprabasal keratinocytes, expressing Foxn1, are key cells in the process of re-epithelialization. The occurrence of an epithelial-mesenchymal transition (EMT) was confirmed by high levels of Snail1 and Mmp-9 expression as well as through co-localization of vimentin/E-cadherin-positive cells in dermis tissue at four days post-wounding. Involvement of Foxn1 in the EMT process was verified by co-localization of Foxn1-eGFP cells with Snail1 in histological sections. Flow cytometric analysis showed the increase of double positive E-cadherin/N-cadherin cells within Foxn1-eGFP population of post-wounded skin cells isolates, which corroborated histological and gene expression analyses. Together, our findings indicate that Foxn1 acts as regulator of the skin wound healing process through engagement in re-epithelization and possible involvement in scar formation due to Foxn1 activity during the EMT process.

## Introduction

Skin, the largest organ of the body, fulfills multiple key functions, acting as environmental protection barrier, an immunological response system and a neuro-endocrine organ [1]. Therefore each injury which interrupts skin integrity has to be immediately repaired to restore its function. Skin repair consists of a series of events, including inflammation, migration, proliferation, granulation tissue formation, and matrix remodeling [2]. These events are synchronized by transcription factors (key molecules in healing process), effector genes and their protein products including cytokines (i.e. Il-1, -6), growth factors (Tgf, Egf, Kgf, Pdgf, Fgf), proteolytic enzymes and their inhibitors (i.e. matrix metalloproteinases—Mmps/Timps), and components of extracellular matrix (collagen, hyaluronic acid) [3, 4]. Whereas effector genes and their protein products have been identified over the past several years, much less is known about the transcriptional regulation of the skin wound healing process.

Transcription factors are key molecules which finely tune gene expression in response to injury and can either promote or suppress gene transcription [3, 4]. AP-1, Pparβ/δ, HoxA3, HoxD3, Smad2 have been identified as regulators of re-epithelialization, whereas fibroplasia and scarring (late stages of wound healing process) appear to be controlled by HoxB13, Smad3 and β-catenin. However none of the transcription factors alone provide a satisfactory explanation of the different pathways and outcomes of skin wound healing resolution: regular scar formation, overgrowing scar tissues formation or non-healing wounds.

There are exceptional mammalian tissues, including the skin of embryos (up to the third trimester of gestation) [5]; the external ears of rabbits, MRL (Murphy Roths Large) [6] and nude mice [7] as well as; digit tips in humans and adult mice [8], which exhibit scarless healing (regeneration). Recently, two models of skin regeneration in adult mammals have been reported, nude [9–11] and *Acomys* [12] mice.

Our study showed that nude (Foxn1 deficient) mice re-grow holes punched in their ears and healed skin injuries in a scar-free/regenerative manner [7, 9–11]. Series of experiments revealed that external ears and skin tissues of nude mice displayed features consistent with a regenerative phenomenon characterized by a blastema formation, (among other related features) [7]. Our next study demonstrated that this regeneration phenomenon was associated specifically with the nude genotype but not with its overall genetic background [7, 9, 10]. Foxn1-deficiency has a pleiotropic phenotype that includes lack of hair, thymus and T- cells. Accordingly, we conducted experiments to determine whether the regeneration phenotype was associated with T-cell deficiency and/or lack of thymus [9]. The studies provided evidence that the skin wound healing process in nude mice displayed characteristic features of regeneration (scar-free healing) that include: (1) fast re-epithelialization, (2) accelerated collagen turnover, (3) high levels of hyaluronic acid expression, (4) low levels of pro-scarring cytokines (Tgfβ and Pdgf), (5) bimodal pattern of Mmp-9 and -13 expression and (6) substantial differences in dermal fibroblasts phenotypes [9–11]. However they revealed that neither lack of thymus or lack of T-lymphocytes explains the regenerative phenotype in nude mice. Since a lack of thymus and T-cells is caused by Foxn1 inactivity in nude mice our attention shifted toward transcription factor Foxn1 as a major factor in the wound healing/regeneration switch.

In nude mice, a nonsense mutation of the *Foxn1* (forkhead box N1) gene, originally described as *Whn* or *Hfh11*, leads to an absence of functional protein [13]. Inactivated Foxn1 in humans and mice results in a lack of hair, immunodeficiency (the absence of thymus and T cells) and skin defects [14, 15]. In humans, nude/SCID (severe combined immunodeficiency) syndrome is characterized by congenital alopecia, functional thymus defect and nail dystrophy [13, 16].

Foxn1 expression is restricted to organs with multilayered epithelia structures such as the thymus and skin. In the thymus, Foxn1 appears to be essential for T cell development [17]. In the skin, Foxn1 expression was detected in the interfollicular epidermal cells and hair follicles [18]. The localization of Foxn1 positive cells in mice skin was present in the first layer of suprabasal cells and in a few basal cells of epidermis [15]. Studies of Brissette group revealed that the Foxn1 expression pattern coincides with the initiation of keratinocytes differentiation showing that both gain and loss of Foxn1 function leads to differentiation abnormalities [14, 15]. Therefore, Foxn1 appears to govern the balance between proliferation and differentiation events that encompass interfollicular epidermal cells and hair follicles [15, 18]. Moreover it has been shown that keratinocytes expressing Foxn1 secrete fibroblast growth factor 2 (Fgf2), which stimulates activation of melanocytes to release pigment, and acting as a mitogen for epidermal cells promoting proliferation of the surrounding epithelium [15, 18].

The sustained balance between proliferation and differentiation of epidermal cells in uninjured skin is perturbed by wounding process.

There are mechanisms and transcription factors which aim to restore integrity of post-wounded tissues and participate in the control of the repair/regeneration processes [19]. One of them is the epithelial-mesenchymal transition (EMT) process. This phenomenon was initially identified during embryonic development. During adulthood, EMT is associated with wound healing and cancer metastasis, processes which may lead to fibrosis and systemic invasion, respectively [20]. During wound healing EMT causes disaggregating and reshaping of epithelia while they acquire mesenchymal phenotype, [21, 22]. Transcription factors like Snail (Snail1), Slug (Snail2), ZEB and Twist are master regulators of EMT [20, 22–25]. Snail family members repress epithelial proteins (like E-cadherin) in cell-to-cell junctions and are balanced by upregulated levels of N-cadherin, which build up mesenchymal connections and exhibit an affinity for extracellular matrix (ECM) [22, 26]. Agents facilitating EMT process via remodeling surrounding ECM and repressing E-cadherin include matrix metalloproteinases (Mmp-2, -9, -13, -14) [22, 26–28]. Among these matrix metalloproteinase 9 (Mmp-9) is well documented to take part in EMT-induced cell migration and invasion [22, 28]. However, despite substantial efforts to examine the EMT process, the mechanism controlling EMT is not completely known.

In this study we investigate the expression of transcription factor Foxn1 during skin wound healing process as a potent factor in reparative wound healing in mammals. This novel data demonstrates that Foxn1 acts as regulator of the skin wound healing process through engagement in re-epithelization and involvement in scar formation due to Foxn1 activity during the EMT process.

## Materials and Methods

### Animals

The present studies were performed on C57BL/6 (B6) and Foxn1::Egfp transgenic female and male mice that were 4–5 weeks old. Foxn1::Egfp transgenic older mice show some abnormalities in hair growth which indicate that introduced transgene may affect skin functionality in older animals. To exclude the possible effect of genetic modification on the experimental outcomes, the study were performed on B6 (qRT-PCR data) and Foxn1::Egfp transgenic mice (Western blot, flow cytometry and immunofluorescent analyses). Foxn1::Egfp transgenic mice [29] were a kind gift from Professor Thomas Boehm (Max-Plank-Institute of Immunobiology and Epigenetics, Freiburg, Germany). Foxn1::Egfp males were crossed to B6 females and F1 mice were analyzed. Mice were bred and housed in a temperature- and humidity-controlled room (22 ± 2°C and 30–70%, respectively) with a 12-h light/12-h dark cycle at the Institute of Animal Reproduction and Food Research of Polish Academy of Sciences, Olsztyn, Poland. Genotyping assays for the Foxn1-eGFP transgene presence were performed for all analyzed animals. DNA was extracted from tail-clipped tissues using the proteinase K (Sigma, MO, USA) and standard phenol-chloroform-isoamyl alcohol DNA (Sigma) isolation protocol. 15 μl PCR reactions were set up with 2 ng genomic DNA (10ng/μl). Primer sequences were: Xah163_F: GTCCCTAATCCGATGGCTAGCTC and NN1_R: GTGCAGATGAACTTCAGGGTC (Genomed, Poland).

### Wound model

The day before the wounding procedure mice were anesthetized, the hair was shaved in dorsal area, and skin was cleaned with Octenisept (Schulke&Mayer, Norderstedt, Germany). The next day, animals were anesthetized by isoflurane and were given four full-thickness wounds made by a sterile 4 mm biopsy punch (Miltex, GmbH, Rietheim-Weilheim, Germany) on the middle of dorsal shaved skin. The surgical procedures were designed to minimize the suffering of experimental animals. After wounding mice were placed in a warmed cage until recovery. Then mice were transferred to individual cages and housed separately for the duration of the study. Experimental animals were monitored daily for any signs of infection with digital photographs documentation starting at day zero and every day for 14 days. Animals were euthanized by CO_2_ asphyxiation at post-injured days 1, 2, 3, 4, 5, 6, 7 and 14 (n = 7–9 per group) and 8 mm diameter skin biopsy punched samples of the surrounded wound area were collected. Wounds and skin tissue collections were conducted at the same time of the day to exclude possible circadian contributions to the outcome of the study. Two skin biopsy punches from each animal were frozen in liquid nitrogen for RNA and protein isolation. The two remaining tissues were fixed in 4% paraformaldehyde (PFA) (Sigma) for cryo-sectioning and 10% formalin (FA) (Sigma) for paraffin sections.

The experimental animal procedures performed in these studies have been approved by the Ethics Committee of University of Warmia and Mazury No. 28/2012.

### Analysis of wound closure and re-epithelialization

The wound healing process was macroscopically monitored and digital photographs were taken at each time point of tissue collection. Wound size [mm^2^] was calculated based on the area of an ellipse = [radius of the length] x [radius of the width] x [π] (number of mice n = 43, number of measurements n = 137) [30]. The percent of wound closure compared to day 0 was calculated.

The re-epithelialization process in Foxn1::Egfp and B6 mice was assessed microscopically on hematoxylin and eosin stained histological slides. Measurements were performed with CellSens Dimension 1 Software (Olympus Soft Imaging Solutions GmbH) and calculated according to the following formula: [length of the extending epidermal tongues]/[length of the wound] x 100% (number of mice n = 18, number of measurements n = 22) [31].

### RNA isolation and quantitative RT-PCR

Total RNA was extracted from skin samples using Tri Reagent (Sigma), while quantity and quality of RNA was checked with NanoDrop1000 (Thermoscientific, MA, USA) and agarose gel electrophoresis. 500 ng of total RNA was reverse transcribed to cDNA using High-Capacity cDNA Reverse Transcription Kit with RNase Inhibitor (Applied Biosystems, MA, USA). We pre-selected housekeeping genes reported as optimal in the wound healing process [32] and after validation: *Tbp* (TATA- binding protein), *Gapdh* (glyceraldehyde 3-phosphate dehydrogenase) and *Hprt-1* (hypoxanthine phosphoribosyl transferase 1), *Tbp* was chosen for normalization as its expression was the most stable. mRNA for *Tbp*, *Foxn1*, *Snail1*, and *Mmp-9* were measured with TaqMan® Gene Expression Assays (Life Technologies, CA, USA). Reactions were performed using 7900HT Fast Real-Time PCR System under conditions: 2 min at 50°C, 10 min at 95°C, 40 cycles 15s at 95°C and 1 min at 60°C. Each run included a standard curve based on aliquots of RNA pool from different skin tissue samples and a non-template control that were analyzed in duplicates. mRNA expression levels were normalized to the reference gene *Tbp* and multiplied by 10.

### Protein isolation and Western blot analysis

Frozen samples of 8 mm skin punches were crushed in liquid nitrogen with a chilled mortar and pestle. Samples were homogenized in 500 μl RIPA buffer containing protease inhibitor cocktail (PhosStop–Roche, Switzerland, Protease Inhibitor–Sigma), and sonicated with Sonics Vibro-Cell ultrasound sonicator (3x20sec, 20kHZ). Protein concentration was measured using the standard Bradford protocol (Bradford Reagent, Sigma). 20 μg of proteins were separated on 12% SDS-polyacrylamide gels and transferred onto polyvinylidene difluoride membranes (Millipore, MA, USA). The membranes were incubated separately with anti-eGFP (1:800, goat polyclonal, AbCam, Cambridge, UK), anti-Foxn1 (1:100, rabbit polyclonal, Santa Cruz; 1:100, goat polyclonal, AbCam) and anti-Gapdh (1:1500, mouse polyclonal, AbCam) antibodies. Then fluorescent secondary antibodies (Alexa Fluor 680, Life Technologies and IRDye 700, Rockland, PA, USA) were applied. Bands were visualized using the Odyssey imaging system (LI-COR Bioscience) according to the manufacturer’s protocol. Densitometric protein analysis was performed as previously described [11]. In the first step we designed a control sample that would be used in each of the Western blot for Foxn1-eGFP protein. Based upon pilot Western blot we have chosen one of post-injured skin sample from Foxn1::Egfp mice as an internal quality control standard to normalize densitometric data from different blots. Four blots were analyzed, which contained n = 4–6 samples per time point. Densitometric analysis was performed using the Odyssey (LI-COR Bioscience) imaging system.

### Histology, immunohistology and immunofluorescent image analysis

Formalin-fixed skin samples were processed, embedded in paraffin and sectioned at 5 μm. Sections were deparaffinized and rehydrated through a graded alcohol series. Slides were stained with hematoxylin and eosin (HE) using a standard protocol. Citra Solution (BioGenex, CA, USA) was used for heat-induced antigen retrieval for eGFP immunohistochemical detection assay. Anti-eGFP antibody (1:200, goat polyclonal, AbCam) staining was performed with the ABC complex (Vectastain ABC kit, Vector Laboratories, Inc., CA, USA). Slides were counterstained with hematoxylin. In control sections, primary antibodies were substituted with non-specific-immunoglobulin G (IgG). Sections were visualized with an Olympus microscope (BX43) equipped with CellSens Dimension 1 Software and photographed with an Olympus digital camera (XC50). Skin tissues for immunofluorescent detection assay were fixed in 4% PFA for 2h, washed in 0.1M PB o/n at 4°C and stored in 18% sucrose/0.1M PB solution prior to cryo-sectioning. Immunofluorescence was performed with the following primary antibodies: collagen IV (1:1000, rabbit monoclonal, Rockland), cytokeratin 16 (1:500, rabbit polyclonal, Novus), E-cadherin (1:1000, rat monoclonal, Invitrogen, CA, USA), Mmp-9 (1:100, rabbit polyclonal, Millipore), Snail1 (1:50, goat polyclonal, Santa Cruz, TX, USA), Snail1 (1:500, rabbit polyclonal, Santa Cruz) and vimentin (1:300, rabbit monoclonal, Abcam). The secondary antibody Alexa Fluor 594 or Alexa Fluor 488 (Life Technologies) were used and nuclei were counterstained with ProLong® Gold Antifade Mountant with DAPI (Life Technologies). In control sections, primary antibodies were substituted with non-specific-immunoglobulin G (IgG). Sections were visualized and photographed with an Olympus microscope (BX43) equipped with an Olympus digital camera (XC50) and analyzed with CellSens Dimension 1 Software (Olympus Soft Imaging Solutions GmbH). Confocal images were scanned and digitalized using an F10i-LIV Laser Scanning Microscope integrated with FLUOVIEW Software (Olympus), with a 60x objective lens. The sequential scans were acquired with Z spacing of 0.5 μm and 1024 x 1024 pixel size at room temperature.

### Flow cytometry analysis

Foxn1::Egfp (n = 6) and B6 (n = 6; control for eGFP specificity signal) mice were used for flow cytometry assay. Mice were sacrificed five days after injury. Four 8 mm samples of injured and an additional four samples of uninjured skin were collected from each mouse. Skin tissues were washed in PBS, minced and digested in 1.23 mg/ml of collagenase (220U/mg) (Life Technologies) for 60 min and filtered through 100, 70 and 40 μm strainers. Cells were centrifuged at 1300 rpm for 9 min. Pelleted cells were re-suspended for 1 min in red blood cell lysing buffer (Sigma) and centrifuged at 1300 rpm for 9 min. Pellets were re-suspended in culture medium DMEM/F12 (Sigma) supplemented with 0,5% fetal bovine serum (FBS, Gibco by Life Technologies) with antibiotics (Penicilin/Streptomycin, Sigma) and counted in Countess TM automated cell counter (Invitrogen). Between 0.3–1 x 10^6^ cells were used for flow cytometric analysis. Side scatter and forward scatter profiles were gated to eliminate dead cells and debris. Detection of Foxn1-eGFP^+^ cells was performed and, within this population, dual staining for epidermal marker (E-cadherin) and dermal marker (N-cadherin) was carried out. Cells were incubated with anti-mouse CD324 (E-cadherin) Alexa Fluor® 647 (BioLegend, CA, USA) and anti-human CD325 (N-cadherin) PerCP-Cy TM5.5 (BD Biosciences, NJ, USA) antibodies and stained with appropriate isotype controls: IgG1 Alexa Fluor® 647 (BioLegend), IgG1 PerCP-Cy TM5.5 (BD Biosciences) for 30 min in the dark at RT. Cells were washed with PBS, and then analyzed with BD FACS Aria II Flow Cytometer running FACS Diva software (BD Biosciences).

### Statistical analysis

Measurements of wound closure, re-epithelialization, quantitative RT-PCR and densitometric data were analyzed using GraphPad Prism, version 6.0 (GraphPad Software Inc.). The means and SEM were calculated for each data set. A one-way analysis of variance (ANOVA) with Tukey’s multiple comparisons tests were used. Flow cytometry data were analyzed for significance using paired Student’s t-test comparing experimental data to the appropriate controls. Statistical significance was set at p-value < 0.05.

## Results

### Macroscopic and microscopic evaluation of the wound healing process

**Fig 1:**
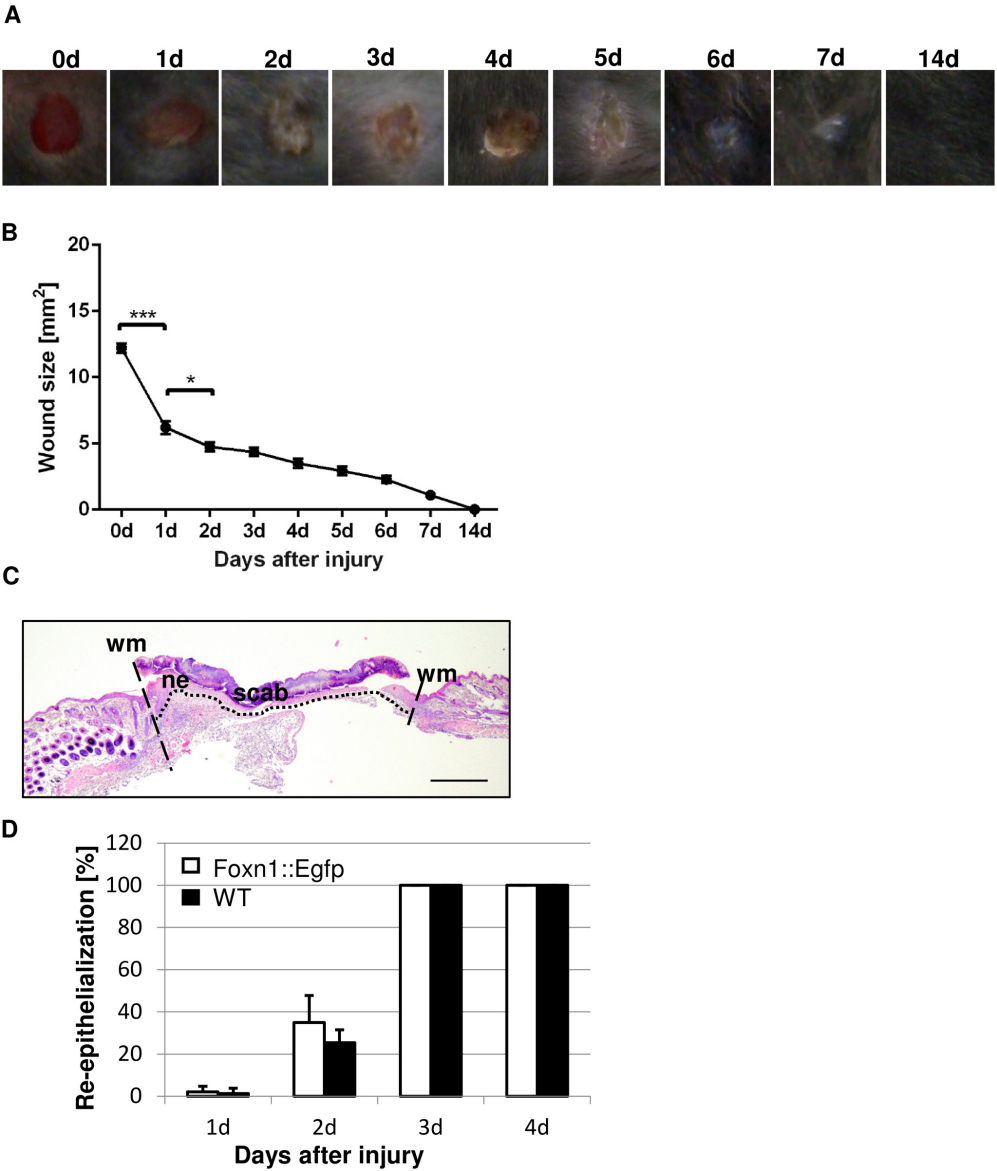
Macroscopic and microscopic evaluation of the skin wound healing process in Foxn1::eGFP and B6 mice. (A) Representative macroscopic views of skin wounds at days 1–7 and 14 after wounding. (B) The morphometrical analysis of the wound closure areas. (C) Representative histological sections of post-wounded skin area of B6 mice at day 3 (hematoxylin and eosin staining; (HE)); scale bar 500 μm. (D) The comparison of time-course of re-epithelialization process between Foxn1::Egfp and B6 mice. wm—wound margin; ne–newly formed epithelium delineated by dotted line. Values are the mean ± SEM; *p<0.05; ***p<0.001.

The macroscopic evaluation of full-thickness excisional wounds on the back of Foxn1::Egfp and B6 mice was performed (Fig 1). The gradual decrease in wound size was detected from day 1 until wound closure was completed at day 14 (Fig 1A and 1B). A significant reduction relative to Day 0 (injury day) in the wound areas was observed at day 1 (p<0.001) and day 2 (p<0.05) after wounding (Fig 1B).

The re-epithelialization process was analyzed on H&E stained microscopic slides separately for B6 (n = 10) and Foxn1::Egfp (n = 9) mice (Fig 1C and 1D). Complete re-epithelialization occurred three days after injury in all animals regardless wild type (B6) or transgenic (Foxn1::Egfp) mice (Fig 1D). No significant differences were observed in the rate of the re-epithelialization process between B6 and Foxn1::Egfp mice (Fig 1D). No infections and complications were reported in any animals during the wound healing experiment.

### Spatial and temporal expression pattern of Foxn1 in the skin wound healing process

**Fig 2:**
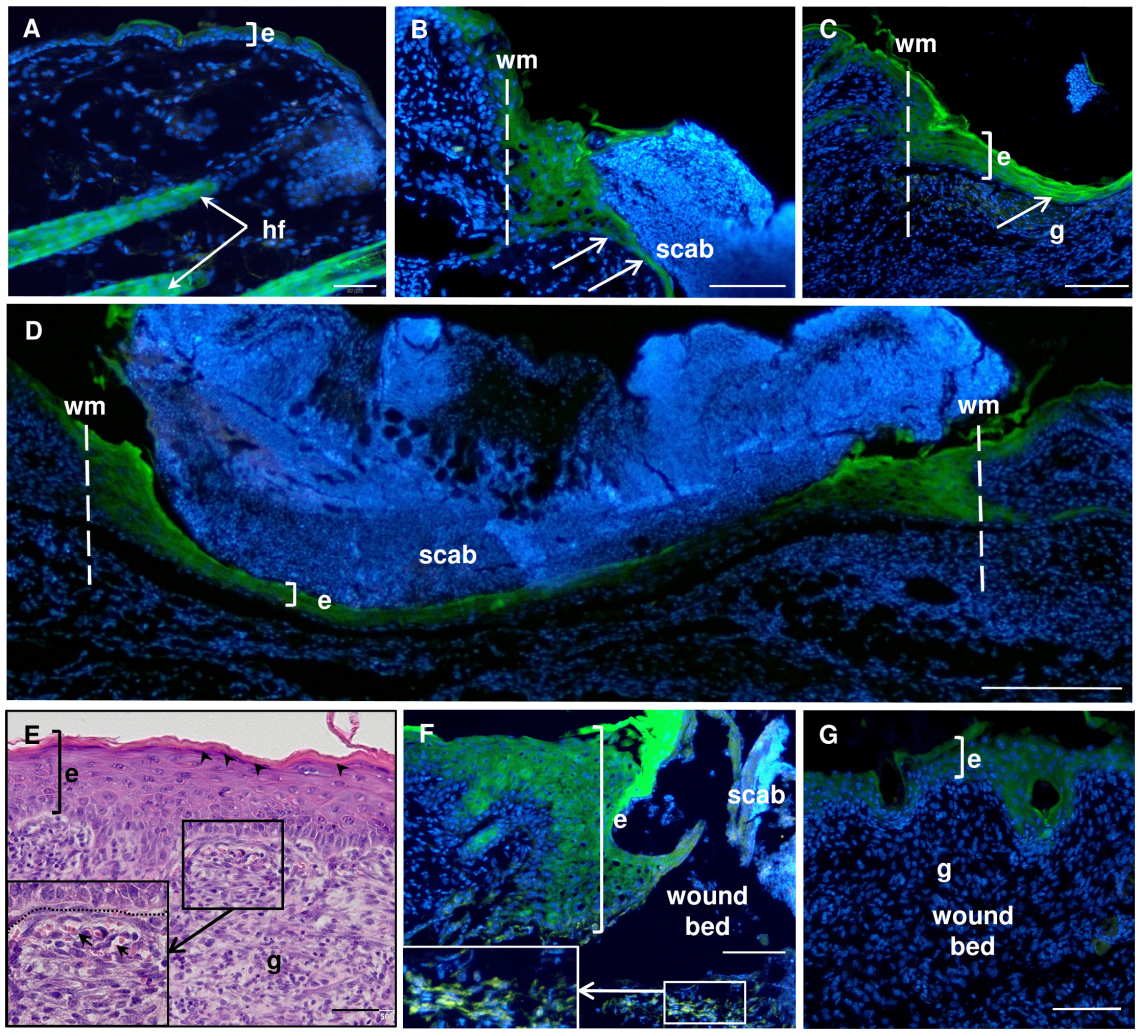
**Spatial and temporal histological analysis of uninjured (A) and post-injured (B-G) skin from Foxn1::Egfp mice.** Skin sections at post-wounded day 2 (B), day 3 (C, D), day 4 (E, F) and day 7 (G). Nuclei were counterstained with DAPI. hf–hair follicle, e–epidermis; g–granulation tissue; wm–wound margin; dotted line delineated basement membrane; arrow–indicates the direction of neo-epithelium migration. Scale bar: 50μm (A, F), 100 μm (B, C, E, G), 200 μm (D).

Analysis of unwounded skin collected from transgenic Foxn1::Egfp mice revealed that Foxn1-eGFP-positive cells localized to interfollicular epidermis and to hair follicles (Fig 2A). In the epidermis, eGFP positivity was detected primarily in the suprabasal and sporadically in basal layers, which is in accordance with previous observations of Foxn1 expression in the skin [15].

Post-wounded skin tissues showed modulation of Foxn1-eGFP signal (Fig 2B–2D and 2F and 2G). Two days after injury, thickened epidermis at the wound margin was built up from Foxn1-eGFP-positive cells (Fig 2B). Intense eGFP signal was also observed in cells forming the leading epithelial tongue migrating underneath the scab (Fig 2B, arrows). Two days after wounding, the neo-epidermis covered, on average, 30% of the wound bed area (Figs 1D and 2B). By the third day, the skin edges were completely bridged by a thin layer of newly formed epidermis (Figs 1D and 2D), which was the thickest at the wound margins. Granulation tissue began to form underneath the epithelium (Fig 2C). Foxn1-eGFP expression was detected at wound margins and Foxn1-eGFP presence was observed on the entire length of neo-epidermis covering the post-wounded area (Fig 2C and 2D).

Multilayered neo-epidermis covering the wound bed showed the presence of flat and squamous cells, indicating functional stratified epithelium at the fourth day after injury (Fig 2E, arrowheads). Granulation tissues formed underneath neo-epidermis were rich in fibroblasts and newly formed blood vessels (Fig 2E inset; arrows). Foxn1-eGFP expression was detected in multilayered neo-epidermis and some Foxn1-eGFP-positive cells were observed in the dermal part of the skin at day 4 (Fig 2F). At day 7, epidermal thickness decreased (Fig 2G). At 14 day post-injury, similar to non-injured skin, the epidermis was formed by only a few layers of Foxn1-eGFP-positive keratinocytes (data not shown). The specificity of eGFP fluorescence was confirmed by an immunohistochemical assay performed on paraffin sections using an anti-eGFP antibody (S1 Fig).

Additionally, to obtain a macroscopic view of the post-wounded skin area, we performed epidermal whole-mounts of the skin (S1 Protocol) collected from Foxn1::Egfp mice at day 5 after injury (S2A Fig) and non-injured skin (S2B Fig). Foxn1-eGFP-positive cells within and adjacent to the wound area were observed (S2A Fig).

**Fig 3:**
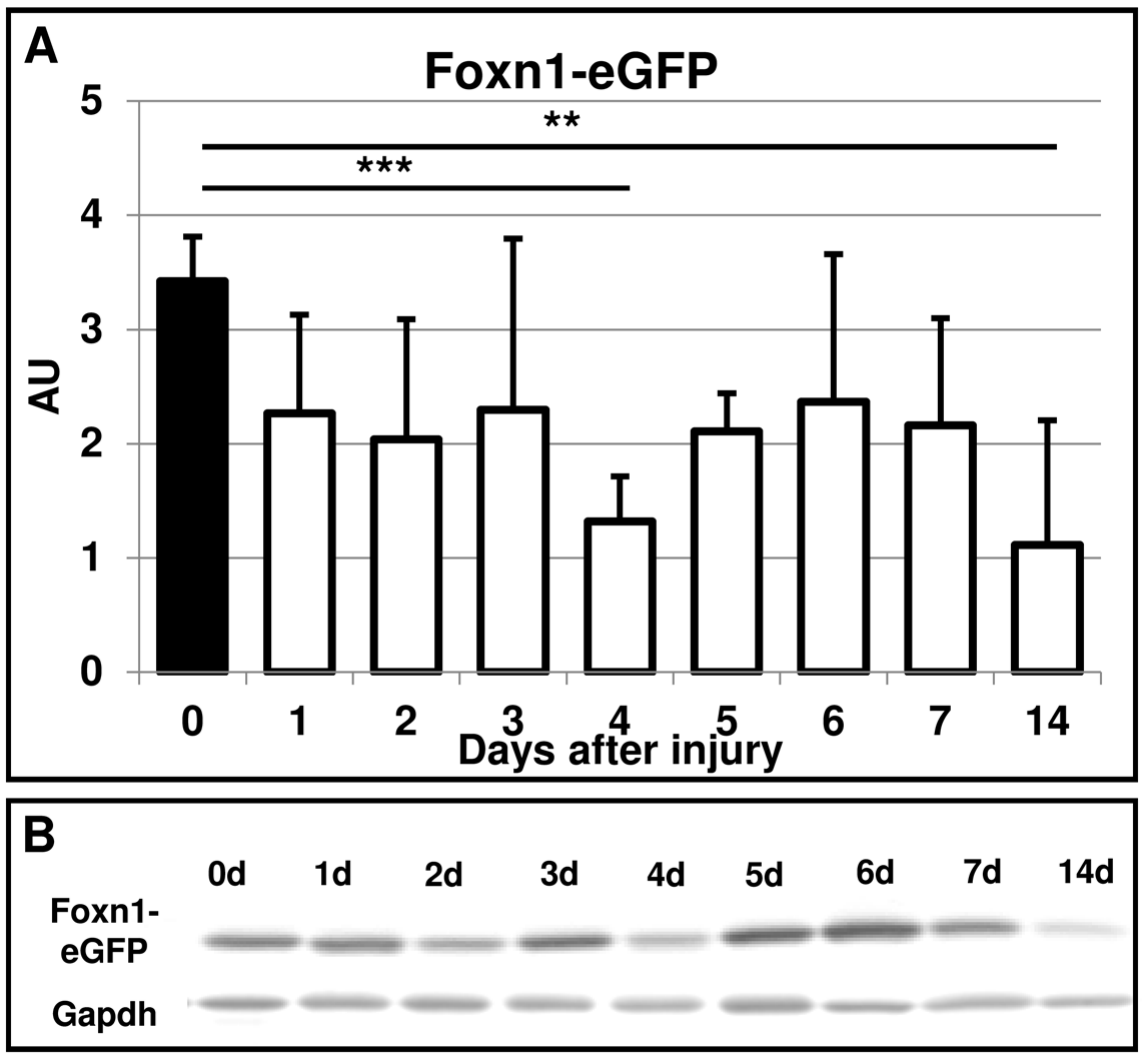
Foxn1-eGFP protein expression during the time course of skin wound healing in Foxn1::Egfp mice. (A) Densitometric analysis of eGFP protein from n = 43 Foxn1::Egfp mice, with n = 4–6 single skin samples per time point. (B) Representative Western blot analysis of Foxn1-eGFP protein expression in skin tissues collected from Foxn1::Egfp mice Values are the mean ± SEM; ** p<0.01; *** p<0.001).

To assess the possible changes in total Foxn1 protein content in skin tissues during the wound healing process, Western Blot analysis was performed. Two types of anti-Foxn1 antibodies were used with inguinal fat tissues serving as a negative control. Unexpectedly, not only skin but fat depot tissues displayed a protein band as a product of Western Blot reaction with molecular weight characteristic for Foxn1 protein (S3A Fig). To estimate the reliability of anti-Foxn1 antibodies we analyzed mRNA collected from matched skin and fat tissues with a Taqman probe to assess *Foxn1* mRNA expression levels. Whereas qRT-PCR assay revealed the reaction product in skin tissues, fat tissues did not show *Foxn1* expression (S3B Fig). These potential false positive results spurred us to determine Foxn1 protein content through an indirect protein analysis. As an alternative approach we performed Western Blot analysis of eGFP protein content collected from post-injured skin tissues of Foxn1::Egfp mice followed by densitometric analysis (Fig 3A and 3B). Post-injured skin tissues were characterized by lower than uninjured controls levels of eGFP. The lowest FOXN-eGFP protein content was detected at post-wounded days 4^th^ and 14^th^ (Fig 3A and 3B).

### Immunofluorescent evaluation of the phenotype of Foxn1-eGFP positive cells

**Fig 4:**
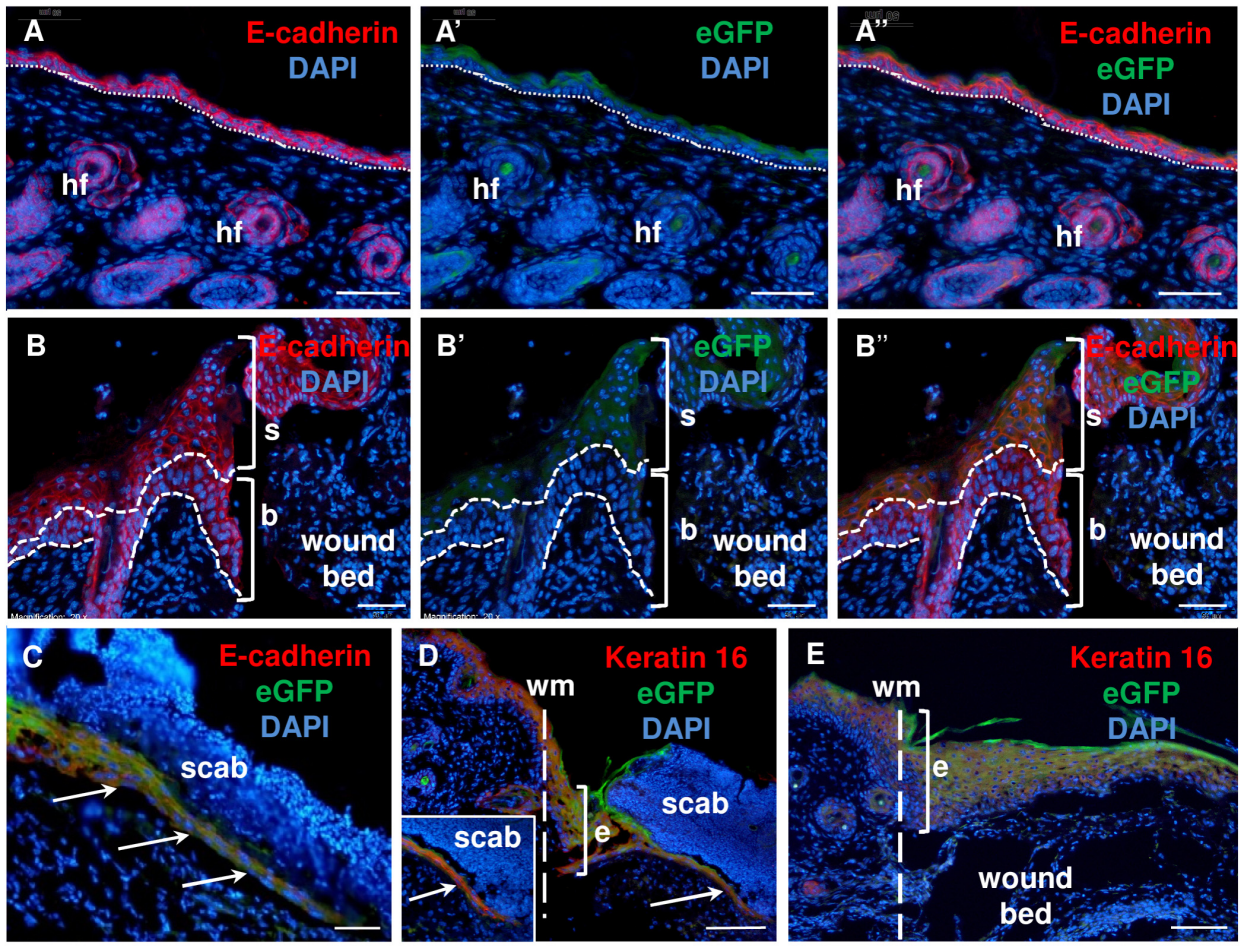
**Immunofluorescent detection of E-cadherin (A–C) and keratin 16 (D–E) expression in uninjured (A) and post-injured at day 2 (B–D) and day 3 (E) skin tissues of Foxn1::Egfp mice.** (A and B) E-cadherin, (A’ and B’) eGFP, (A” and B”) co-localization of E-cadherin and eGFP. hf–hair follicles; wm–wound margin; dotted line–basement membrane (a, a’ and a”); dashed lines–delineated epidermal basal cells (b, b’ and b”); arrow–neo-epithelium migration; e–epidermis; b–basal epidermal layer; s–suprabasal and squamous epidermal layers. Scale bar 50 μm (A-A”, B-B” and C); 100 μm (D-E).

To determine the phenotype and spatial profile of Foxn1-positive cells in post-injured skin, double fluorescent imaging was performed on histological sections from Foxn1::Egfp mice together with fluorescent antibodies for: E-cadherin, cytokeratin 16 (K16) or vimentin. E-cadherin (an epidermal marker) deposition was revealed in the entire epidermis of non-injured (Fig 4A) and post-injured skin, including thickened wound margin (Fig 4B). Foxn1-eGFP signal localized to the suprabasal layer, where it co-localized with E-cadherin, but not the basal layer of epidermis (Fig 4A’ and 4B’). Interestingly, the double E-cadherin/eGFP staining revealed a thick multilayered basal epidermal part at the wound margin as compared to a single layered basal epidermal part in non-injured skin. (Fig 4A, 4A” and 4B”). Contrary to the thickened epidermal at wound margin (Fig 4B, 4B’ and 4B”), the leading epidermal tip showed co-localization of Foxn1-eGFP and E-cadherin expression along its entire extent and thickness (Fig 4C).

To assess whether eGFP expression in the epidermal tongue was associated with activation in response to the skin injury state of keratinocytes, we determined the co-localization of K16, as a marker uniquely expressed in the suprabasal layer of the epidermis [33]. It was determined that every single cell comprising the leading epidermal tongue showed Foxn1-eGFP and K16 co-localization (Fig 4D, inset). However, the epidermal wound margin (Fig 4D) and newly formed epidermis that covered the entire wound bed (Fig 4E) showed co-localization of eGFP and K16 signal within the epidermis except basal keratinocytes layer.

### Estimation of Foxn1 contribution to EMT during the skin wound healing process

**Fig 5:**
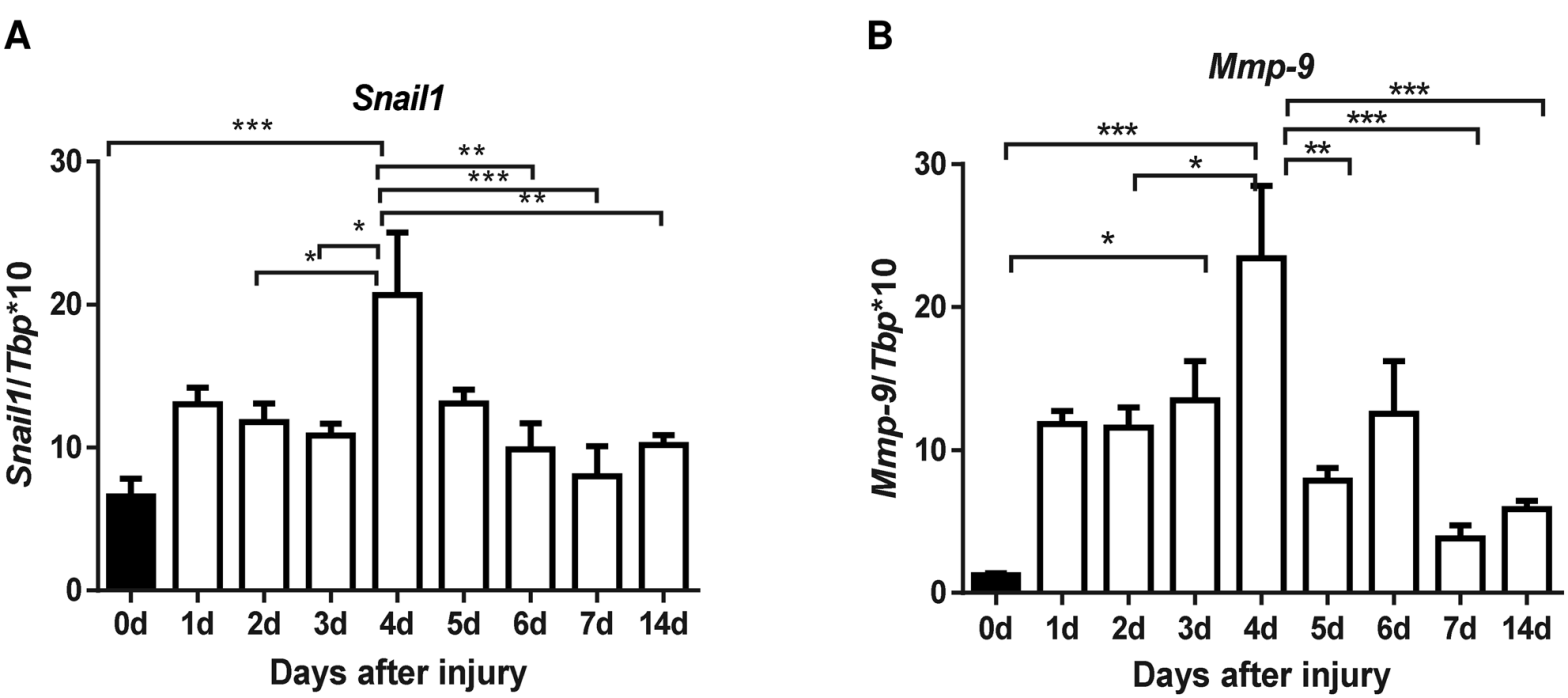
***Snail1* (A) and *Mmp-9* (B) mRNA expression during the time course of skin wound healing in B6 mice.** Expression of *Snail1* (A) and *Mmp-9* (B) mRNA was analyzed in single skin samples (n = 6–9 per time point) and were normalized by the levels of *Tbp* mRNA. Values are the mean ± SEM; * p<0.05; ** p<0.01; *** p<0.001).

Snail1 and Mmp-9 have been identified as markers of EMT [27]. Analysis of mRNA expression levels for *Snail1* and *Mmp-9* during skin wound healing are shown in Fig 5A and 5B, respectively. The highest *Snail1* and *Mmp-9* expression levels in post-injured skin tissues were detected at post-injured day 4^th^ (Fig 5A and 5B).

**Fig 6:**
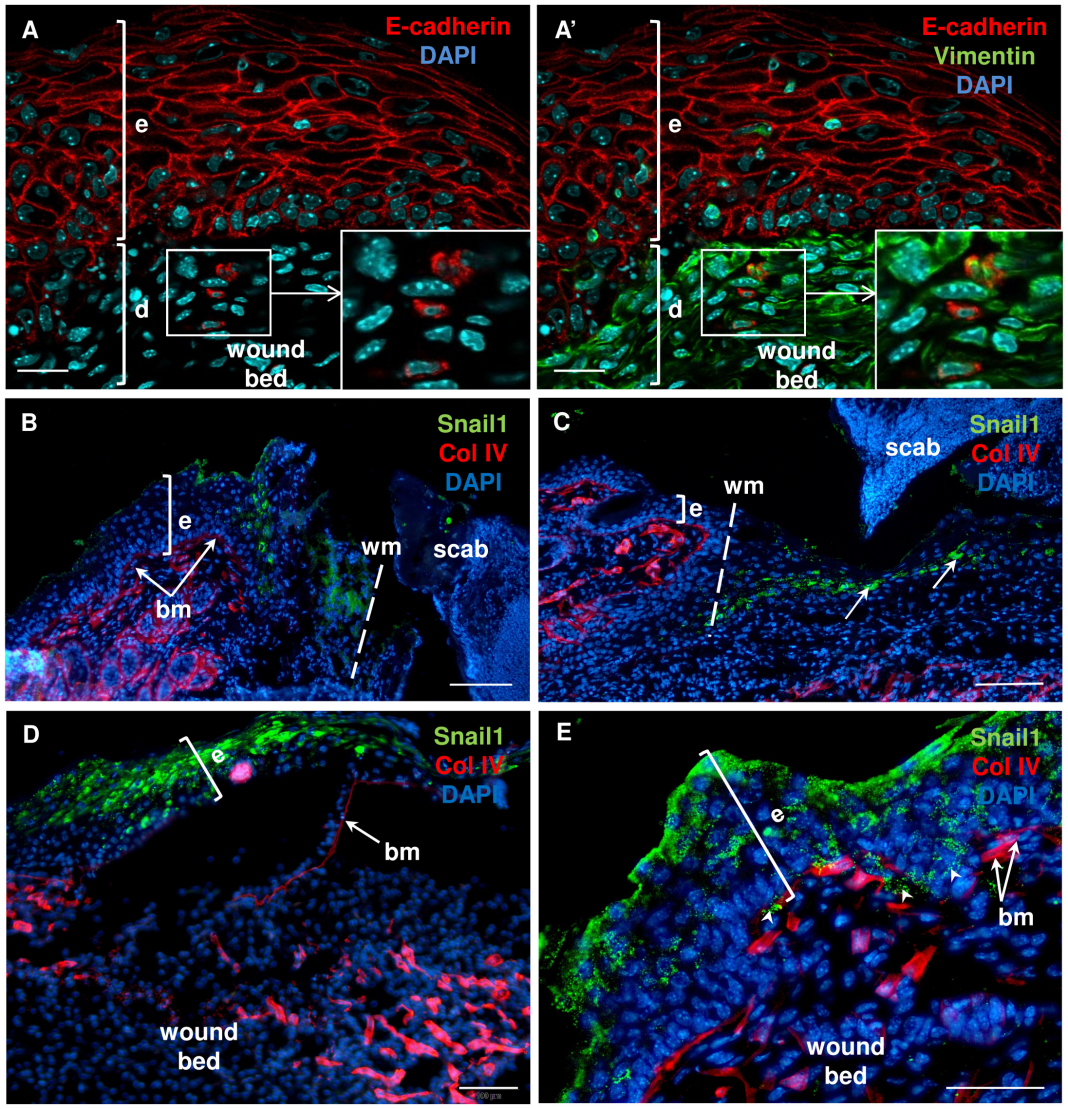
Immunofluorescent detection of EMT traits during the skin wound healing process in B6 mice. Confocal microscopy imaging (A and A’) of co-localization of E-cadherin and vimentin-positive cells at day 6 after injury; insets provide its higher magnification. Immunostaining for Snali1 and Col IV were detected at post-wounded days: 2 (B), 3 (C), 4 (D), and 5 (E); nuclei were counterstained with DAPI. hf–hair follicle; e–epidermis; d–dermis; wm–wound margin; bm/arrows at B, D and E–basement membrane; arrowheads–Snail1 positive cells between fragmented basement membrane. Scale bar 20 μm (A-A’), 50 μm (E) and 100 μm (B-D).

To evaluate the possible presence of the EMT phenotype cells in skin tissues during wound healing, we performed immunofluorescent staining for epidermal (E-cadherin) and dermal (vimentin) markers. The presence of fibroblast-like cells showing co-localization of E-cadherin and vimentin was detected in the dermal part of the skin in proximity to the epidermis at days 4–6 after wounding (Fig 6A and 6A’). Next, we applied anti-Snail1 and anti-collagen IV (Col IV provides the structural framework of the basement membrane) antibodies to determine the localization of the Snail1-positive population of cells, in relation to disrupted basement membrane during wound healing (Fig 6B–6E). At days 2 and 3 after wounding, there were populations of Snail1-positive cells in the epidermis at the wound margin (Fig 6B) and within the epithelial leading tongue (Fig 6C, arrows). The presence of thin, immature, and non-continuous basal lamina beneath the new epidermis was revealed at day 4 (Fig 6D) and day 5 (Fig 6E) after injury. At day 4, immunostaining for Snail1 revealed abundant Snail1 positivity in areas of hyper-proliferating epidermis in the center of the wound bed (Fig 6D). At day 5, Snail1 expression was observed in the newly formed epidermis and in the cells localized between fragmented structures of the basal lamina (Fig 6E, arrowheads).

**Fig 7:**
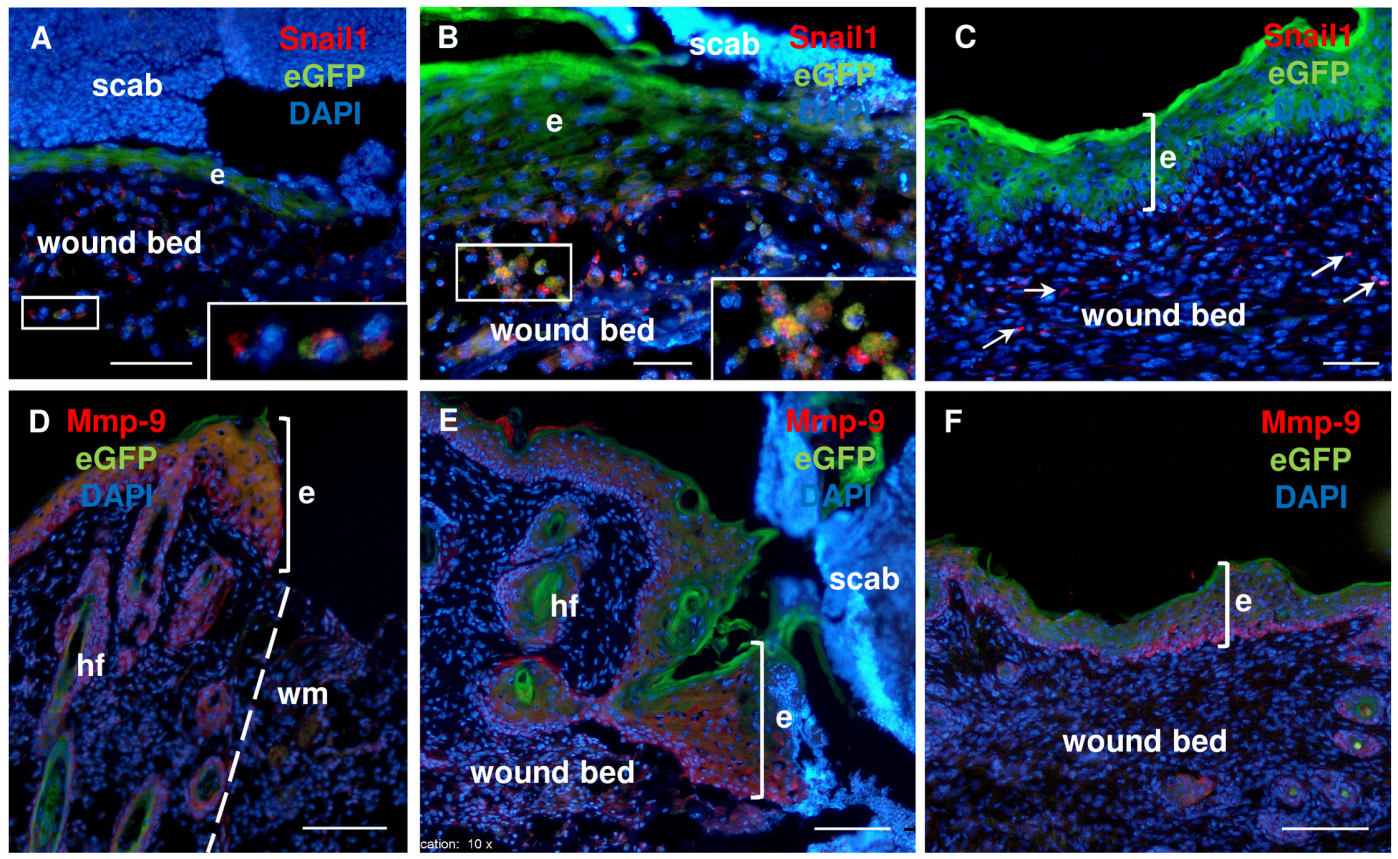
Fluorescent detection of Foxn1-eGFP and EMT markers during skin wound healing process in Foxn1::Egfp mice. Foxn1-eGFP and Snail1 fluorescent detection at days: 2 (A), 4 (B), and 6 (C). Foxn1-eGFP and Mmp-9 fluorescent detection at days: 2 (D), 4 (E), and 6 (F). Nuclei were counterstained with DAPI. hf–hair follicle; e–epidermis; wm–wound margin; arrows–Snail1 positive cells in dermis; insets show higher magnification. Scale bar 50 μm (A-C), 100 μm (D-F).

Next, to examine whether Foxn1-eGFP-positive cells contribute to the EMT process, fluorescence imaging of post-wounded skin tissues from Foxn1::Egfp mice with Snail1 staining as an EMT marker was performed (Fig 7A–7C). Snail1-positive, interspersed single cells were detected in the dermal part of the wound bed after 2 days post-injury (Fig 7A). Some of them co-localized with eGFP-carrying cells (Fig 7A, inset). The population of Foxn1-eGFP and Snail1 co-expressing cells was detected at days 4 and 5 in the dermal part of post-wounded skin (Fig 7B). At day 6, the number of cells expressing Snail1 decreased to a few cells interspersed within the dermal part of the wound bed (Fig 7C).

The second marker of EMT process, Mmp-9, revealed its presence in all layers of the epidermis and in hair follicles (Fig 7D–7F). Mmp-9 co-localized with epidermal Foxn1-eGFP signal and was also present in the eGFP-free basal layer of epidermis (Fig 7D–7F). Robust expression of Mmp-9 at post-wounded day 2 was observed in the thickened epidermis at the wound margin, which co-localized with Foxn1-eGFP fluorescent signal (Fig 7D). Abundant Mmp-9 staining was detected at day 4 after injury in hyper-proliferative epidermis and in the dermis within the wound bed (Fig 7E). At day 6, Mmp-9 expression was reduced, although some Mmp-9 staining was observed in the basal layer of the epidermis and there were single Mmp-9-positive cells interspersed in the dermal part of the wound bed (Fig 7F).

**Fig 8:**
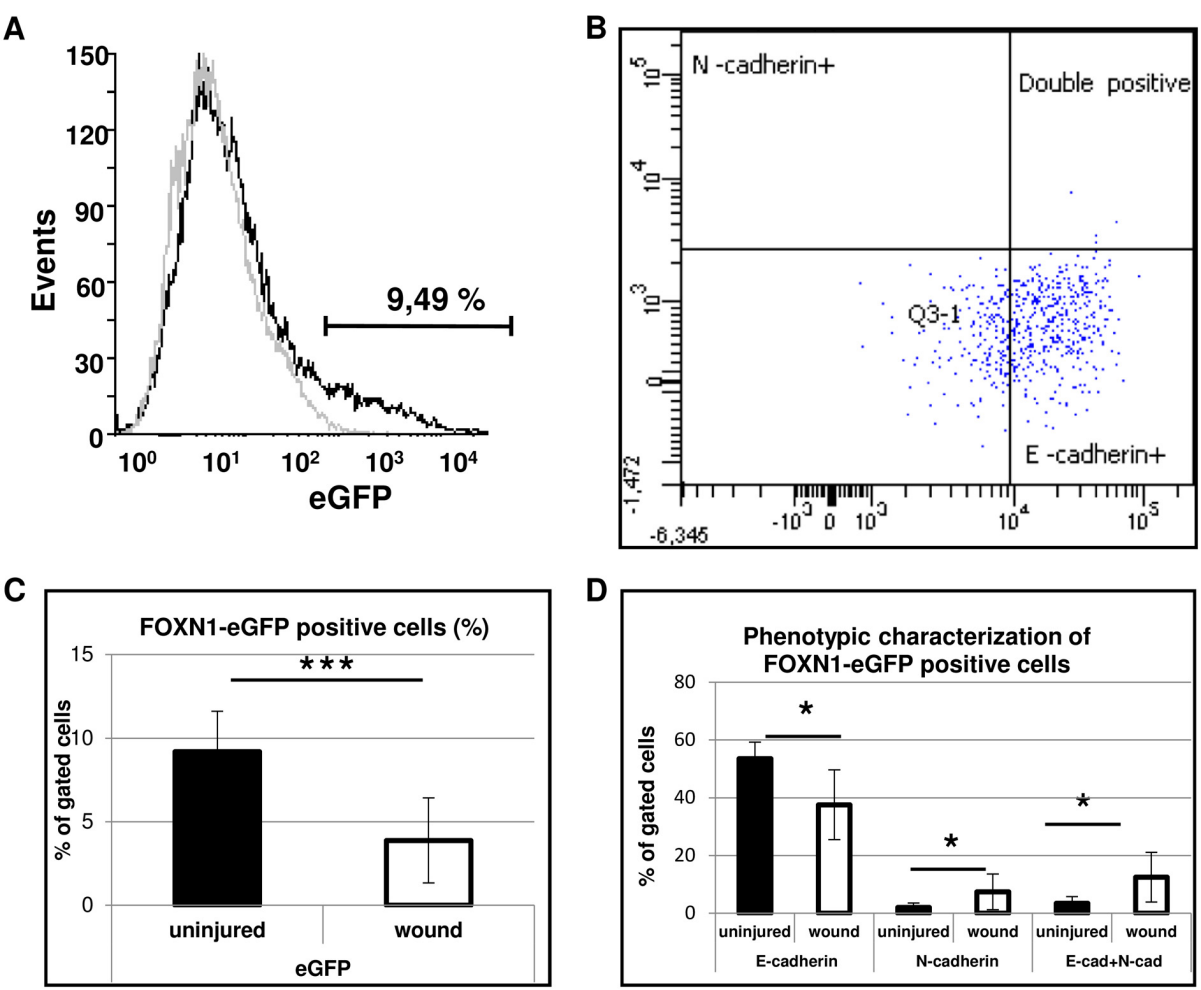
Flow cytometry analysis of cells isolated from post-injured and uninjured skin area of Foxn1::eGFP mice at post-wounded day 5^th^. (A) Representative FACS histograms show detection of Foxn1-eGFP-positive populations of cells from injured skin of Foxn1::Egfp (black) and B6 (grey) mice. (C) Foxn1-eGFP-positive population of cells in uninjured versus injured skin samples. (B, D) Analysis of E-cadherin and N-cadherin positive cells within Foxn1-eGFP population. Data represents mean ± SEM; n = 6.

Fluorescence-activated cell sorting (FACS) analysis was used to establish the phenotypic (epidermal/dermal) status of Foxn1-eGFP-positive cells during skin wound healing (Fig 8). Wild type mice were used as a negative control for eGFP signal (Fig 8A). Cells isolated from uninjured and post-injured skin areas of Foxn1-eGFP mice at post-injured day 5 were stained with anti-E-cadherin and anti-N-cadherin fluorescent antibodies (Fig 8B). Gating with forward scatter (FSC) versus site scatter (SSC) was used to define the dominant population of the analyzed skin cells.

There was a significant decrease of Foxn1-eGFP-positive cells at day 5 after injury, from 9.2% to 3.8% (p<0.001; Fig 8C). The analysis of Foxn1-eGFP-positive cells revealed that the majority of eGFP-positive cells showed E-cadherin positivity with a difference between uninjured (53.56%) vs. post-injured (37.58%) skin cell isolates (Fig 8D). Foxn1-eGFP/N-cadherin-positive cells comprise 1.98% of cells in uninjured samples and a significantly higher 7.4% of cells isolated after injury. Intriguingly, within the Foxn1-eGFP population, we observed cells positive for both E-cadherin and N-cadherin (Fig 8D). The population of E-cadherin/N-cadherin-positive cells increased from 3.41% detected in uninjured skin tissues to 12.5% at day 5 after injury (Fig 8D). Those quantitative data together with qualitative detection of Foxn1-eGFP+Snail double positive cells support Foxn1 participation in skin wound healing through EMT process.

## Discussion

**Fig 9:**
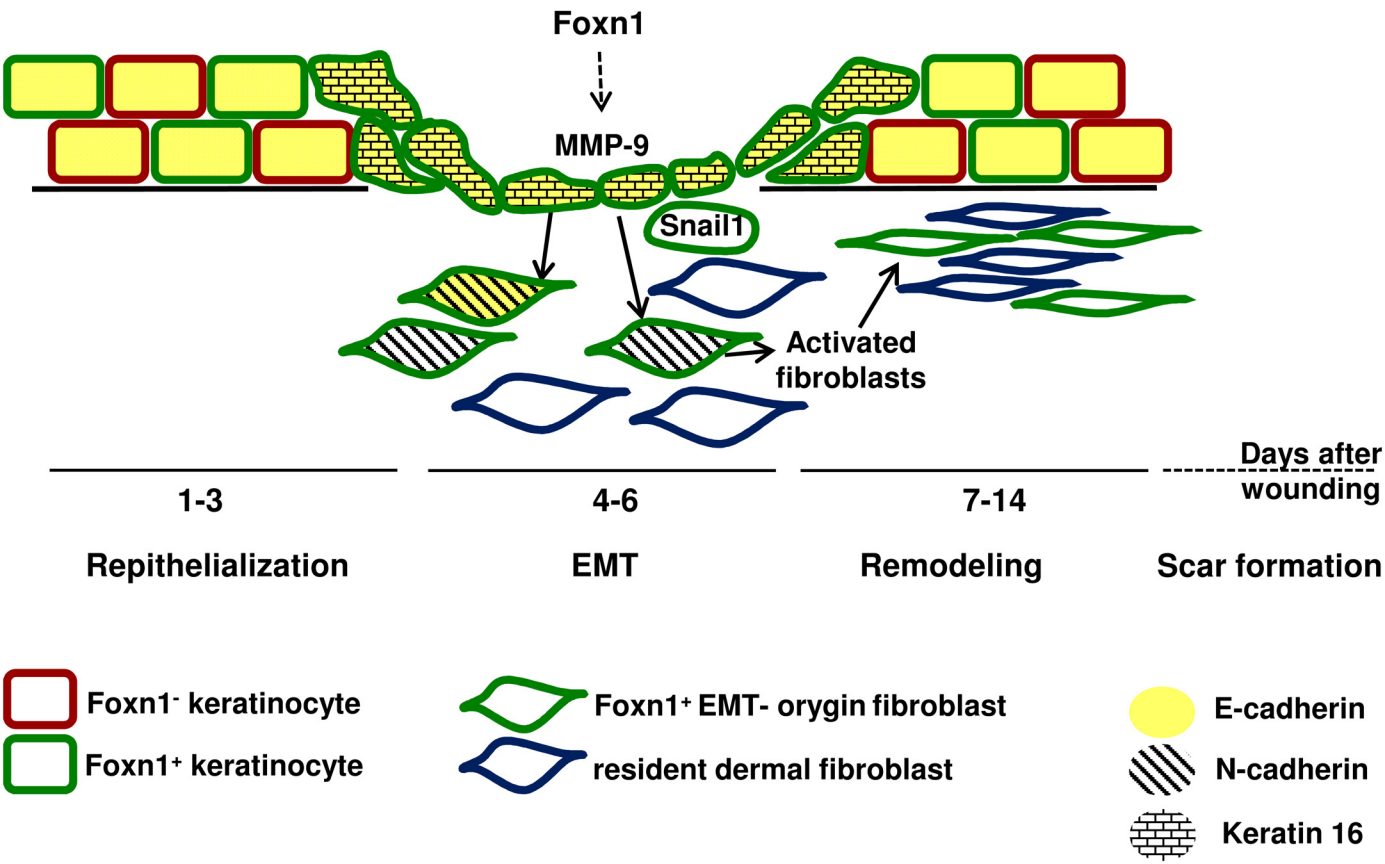
Schematic illustrating the proposed role of Foxn1 in scar forming through involvement in EMT process.

Healing by fibrosis, instead of regeneration, causes a huge problem in public health. Scar formation which takes place after trauma, surgery, or burn usually results in a decrease in the functionality and the cosmetic appearance of the skin. Moreover, scar formation that occurs in the skin generally leads to tissue dysfunction. Thus, prevention or reduction of scarring remains one of the major goals of medical research. Accordingly, understanding the mechanism of scar formation will expand our knowledge of skin injuries and provide novel therapeutic targets. Skin wound healing is a coordinated and rapid physiological process under the control of a magnitude of factors, including transcription factors. The present data revealed that the transcription factor Foxn1 acts as a regulator of skin wound healing. We showed that during the skin wound healing process, Foxn1 is modulated and, together with K16, participates in re-epithelization and may contribute to scar formation due to activity in the EMT process (Fig 9). In light of our previous findings showing that Foxn1 deficient (nude) mice exhibit scar-free skin healing, we propose that Foxn1 activity/inactivity acts as major switch between reparative (scar forming) and regenerative (scar free) healing (see Fig 1 in *Gawronska-Kozak et al*., *2014*) [34].

Fluorescent analysis of post-injured skin tissues collected from transgenic Foxn1::Egfp mice revealed Foxn1 expression in the multilayered suprabasal and squamous part of the epidermis adjacent to the wound margin. Moreover, post-injured skin tissues showed multiplication of both suprabasal (Foxn1-positive) and basal (Foxn1-negative) cells, which could indicate a dual effect of Foxn1 on post-wounded tissue. One interpretation of these findings is that Foxn1 directly impacts keratinocytes differentiation in repaired epidermis and stimulates the proliferation of basal cells due to a paracrine effect. As Foxn1 is considered to be a factor responsible for proliferation of basal keratinocytes, the transition from basal to differentiating suprabasal cells [14] and the promotion of early stages of differentiation [15], this supports the hypothesis that Foxn1 up-regulation after injury may have an active and complex role in the process of skin wound healing. Further studies using skin cell lineage specific Foxn1 knock-out models will be a necessary future experiment to explore this intriguing hypothesis. The laboratory is exploring the acquisition of floxed Foxn1 mice and appropriate keratinocyte promoter Cre strains for this purpose.

Re-epithelialization, a critical initial step in the skin wound healing process, is achieved by migration/proliferation of keratinocytes surrounding the wound bed area [2]. There are two main theories about motile activity of keratinocytes during the re-epithelialization process that differ with respect to the role of suprabasal vs. basal cells as initiators of this process [33, 35]. Recently, Safferling and colleagues [36] showed that basal cells migrate into the wound region, lift suprabasal cells and form a multilayered tongue, creating a compartment composed of basal and suprabasal cells. Our fluorescent detection of Foxn1-eGFP showed its association with suprabasal keratinocytes at the wound margin and in post-wounded skin epithelium. Unexpectedly, we observed that each cell of the migrating epithelial tongue expressed Foxn1-eGFP co-localized with K16 expression. It has been shown that K16-positive keratinocytes are part of the activated, hyperproliferative epithelia and are present in the leading tip during re-epithelialization [37]. Since inducible keratin K16 is expressed in the suprabasal layer in the post-wounded skin wound area [33] and co-localizes with Foxn1-eGFP cells, our data support the concept that suprabasal keratinocytes, expressing Foxn1, are key cells in the process of re-epithelialization. Moreover, the data by Prowse et al. showing that overexpression of Foxn1 in the skin of transgenic mice stimulates expression of keratin 6 (partner of K16) support our results [14]. The present data implicate Foxn1 as a transcription activator contributing to the activation of keratins (i.e. K16) required for epithelial resurfacing of post-wounded skin tissues. EMT is a developmental program in which epithelial cells acquire a fibroblast-like morphology. EMT is often listed as a phenomenon taking part in the skin wound healing process, although its exact impact remains elusive. During skin wound repair, EMT can be considered in two aspects: (1) re-epithelialization when activated keratinocytes acquire migratory capacity (mesenchymal phenotype) [35] and (2) supplying dermis with activated fibroblasts and myofibroblasts from the pool of epithelial cells [24]. Re-epithelialization is thought as not complete process of EMT although it appears to be orchestrated by similar growth factors, transcription factors, and intracellular signaling pathways characteristic of developmental EMT [26, 38, 39]. A well-known feature of the EMT process during the acquisition of the mesenchymal phenotype is the loss of keratins and the gain of mesenchymal markers like vimentin, N-cadherin and FSP-1 [35]. It is reported that during re-epithelialization, keratinocytes lose some junctional connections and move as compartments, while they do not express vimentin and N-cadherin [26, 38]. In our studies, we observed populations of Snail1-positive cells migrating as compartments during re-epithelialization at the wound margin and expanding into the epithelium covering the wound bed. One of the characteristic features of EMT changes is the expression of inducible keratins reported during developmental EMT [35] and in hypertrophic skin [40]. Our studies showed K16 expression present in the epithelium of post-wounded tissue. The fact that all K16-positive cells simultaneously expressed Foxn1 point to a contribution of this transcription factor in the EMT changes.

The epidermal response to injury is followed by a wave of dermal repair [41]. Fibroblasts represent heterogeneous populations of skin cells and it has been recently reported that only restricted cell lineages mediate scar formation [41]. In general, fibroblasts within the wound bed can arise from distinct sources by proliferation and migration from unwounded adjacent tissue [42], bone marrow [43] and through EMT [24, 25]. Fibroblast-like cells (FSP-1- and/or vimentin- expressing keratinocytes) were observed in the epidermis and an increased number of FSP-1-positive cells were present within the dermal part of scar tissues after thermal injury [24]. Our present confocal microscopy data has revealed cells with a transient epidermal-dermal phenotype (E-cadherin and vimentin co-localization) interspersed within the dermis (see Fig 6A and 6A’). It has been stressed that visualization of cells undergoing EMT is rare, since epithelial cells rapidly lose expression of epidermal markers after transition and it is not possible to detect the transient phenotype [44]. EMT is much more often visualized in cancer models. It was acknowledged that in cancer tissues, growth factors and signaling molecules inducing EMT act continuously for a much longer time and enable easier detection of transient, metastable cells than is possible during the rapid process of skin wound healing [38]. Franci et al. studying the skin wound healing process showed increased expression of Snail1-positive cells within the dermis, with the highest number of scattered Snail1-positive cells during re-epithelialization [23]. In our studies, up-regulated EMT markers, *Snail1* and *Mmp-9*, were present at four days after wounding, while Snail1-positive keratinocytes crossing the broken basement membrane during wound healing and the presence of E-cadherin/vimentin-positive cells in dermis at days 4–6 clearly suggest that EMT occurred. Moreover, double fluorescent detection enabled us to observe Snail1-positive, migrating single cells in the dermal part of the wound bed that co-localized with Foxn1-eGFP bearing cells. Additionally, quantitative measurements of E-cadherin and N-cadherin surface markers within Foxn1-eGFP^+^ cells by a flow cytometry assay revealed a population of cells with transient E-cadherin^+^/N-cadherin^+^ phenotype in post-injured tissue, which could indicate an active EMT process during skin wound healing. This observation was made five days after wounding when the re-epithelialization process was already completed. This suggests Foxn1 participation in the EMT process and indicates that post-wounded days 4–6 of healing is an essential step in the EMT process. Furthermore, the days 4–6 may be a critical time for possible intervention aiming to switch reparative (scar present) vs regenerative (scar free) skin wound healing resolution. Snail1 action is connected with repression of the epidermal and activation of the mesenchymal phenotype, changes in its subcellular localization and regulation of EMT target genes [22]. Since Snail1 is an inducer of EMT, and Foxn1 is regarded as a transcriptional activator, synergy between these two factors is a potential contributor to the EMT process.

The molecular mechanisms underlying the transcriptional pathway of Foxn1 affecting epithelial plasticity and epidermal-dermal cross-talks are not completely recognized. It has been shown that Foxn1 is part of the Notch-Wnt regulatory axis controlling hair follicle function, which encompasses epithelium and adjacent dermis [45]. Foxn1 has been reported as a differentiation factor in hair follicles together with Msh homeobox (*Msx2*) gene function in a parallel pathway [46]. It was revealed that Msx2 controls epithelial morphogenesis, and is involved in the skin wound healing process. Interestingly, *Msx2* null mice exhibit faster re-epithelialization [47], similar to reported accelerated wound closure in Foxn1-deficient nude mice [11].

Our previous data showed that Foxn1 inactivity in nude mice creates pro-regenerative conditions accountable for scar-free skin healing when injury occurs. The present analysis indicates that Foxn1-bearing cells participate in the wound healing process through engagement in re-epithelization and possible involvement in scar formation due to Foxn1 activity during the EMT process. This data can provide insights into novel therapeutic targets in wound healing management. The localization of Foxn1 expression to the skin and thymus can prove to be beneficial by providing a tissue specific transcription factor as a target for future medical treatments.

## Supporting Information

S1 Fig Immunohistochemical assay with anti-eGFP antibodies performed on post-wounded skin sections of Foxn1::Egfp mice. e–epidermis; wm–wound margin marked by dashed line; hf–hair follicles. Scale bar 100 μm (A), 50 μm (B).
(TIF)

S2 Fig **Whole mounts dorsal epidermis from Foxn1::Egfp mice of postinjured (A) and uninjured (B) skin area at day 5 post-injury.** Arrows indicate wound margin. Scale bar 500 μm (A) and 50 μm (B).
(TIF)

S3 Fig **Detection of Foxn1 protein with Western Blot (A) and *Foxn1* mRNA with qRT-PCR (B) analysis.** False positive Foxn1 protein band present in inguinal fat and skin tissues (A). Expression of *Hprt1* mRNA in skin and inguinal fat tissues, and *Foxn1* mRNA in skin tissues (B).
(TIF)

S1 Protocol Epidermal whole-mounts protocol. Foxn1::Egfp (n = 3) and B6 (n = 1; negative immunofluorescent control) animals were used for whole mount slide analysis. 8 mm diameter post-injured and uninjured skin tissues were collected, washed in PBS, incubated overnight in 3.33 mg/ml dispase enzyme (1.89U/mg) (Life Technologies) in Hanks' balanced salt solution (HBSS; Sigma) at 4°C. Then, skin samples were placed in pre-warmed (37°C) 0.05% trypsin-EDTA solution (Trypsin-EDTA 1x, Life Technologies) for 10 min. Epidermis were separated from the dermis as an intact sheet and was placed on glass slides fixed in 4% PFA for 20 min, washed in PBS and mounted in ProLong® Gold Antifade Mountant with DAPI (Life Technologies). Sections were visualized with an Olympus microscope (BX43) and photographed with an Olympus digital camera (XC50).
(DOCX)
