# Supplementary figures and images for: Foxn1 Transcription Factor Regulates Wound Healing of Skin through Promoting Epithelial-Mesenchymal Transition
Source: PLoS One. 2016 Mar 3;11(3):e0150635. doi: 10.1371/journal.pone.0150635 (PMC4777299; doi:10.1371/journal.pone.0150635)

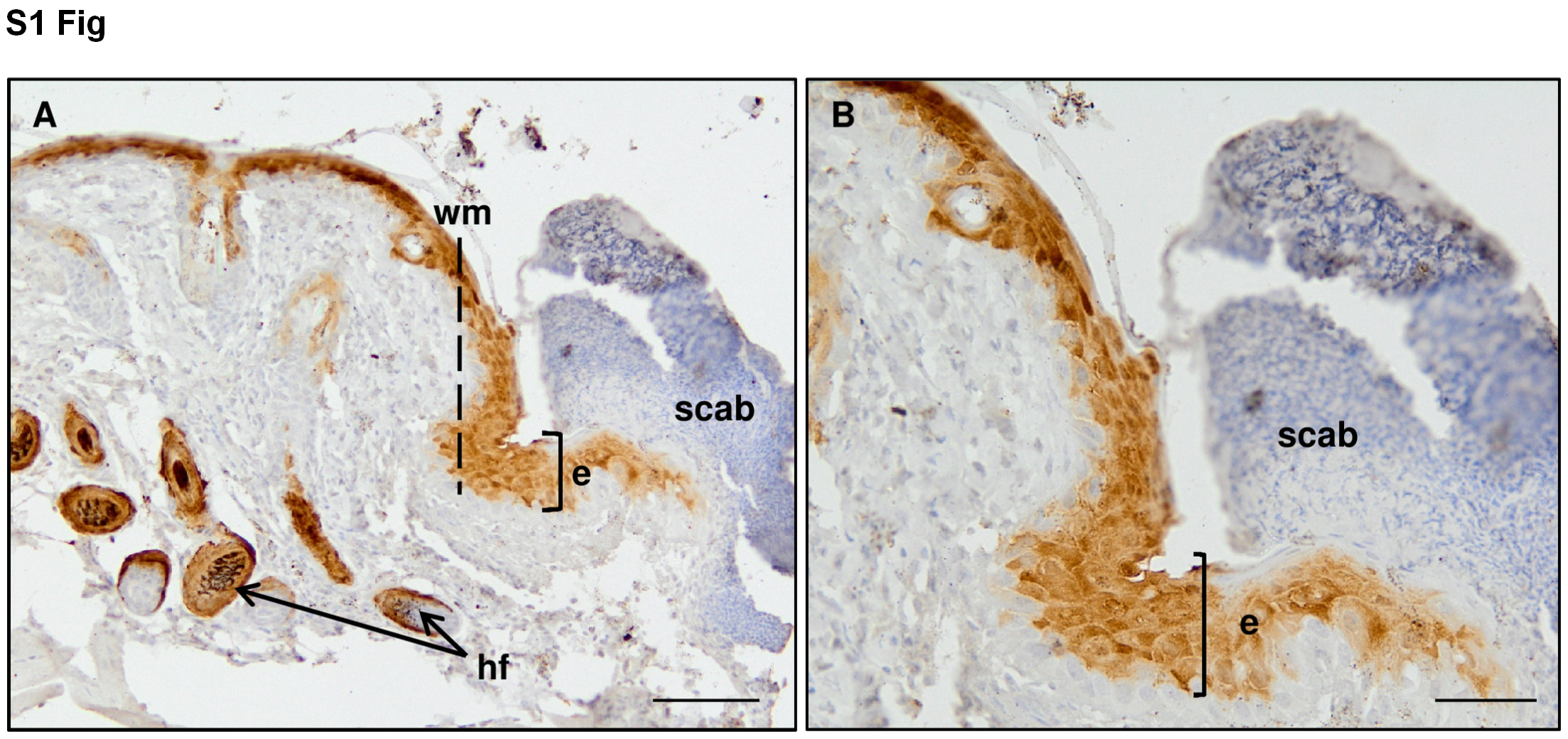

Supplement: S1 Fig — e–epidermis; wm–wound margin marked by dashed line; hf–hair follicles. Scale bar 100 μm (A), 50 μm (B). (TIF) [file pone.0150635.s001.tif]

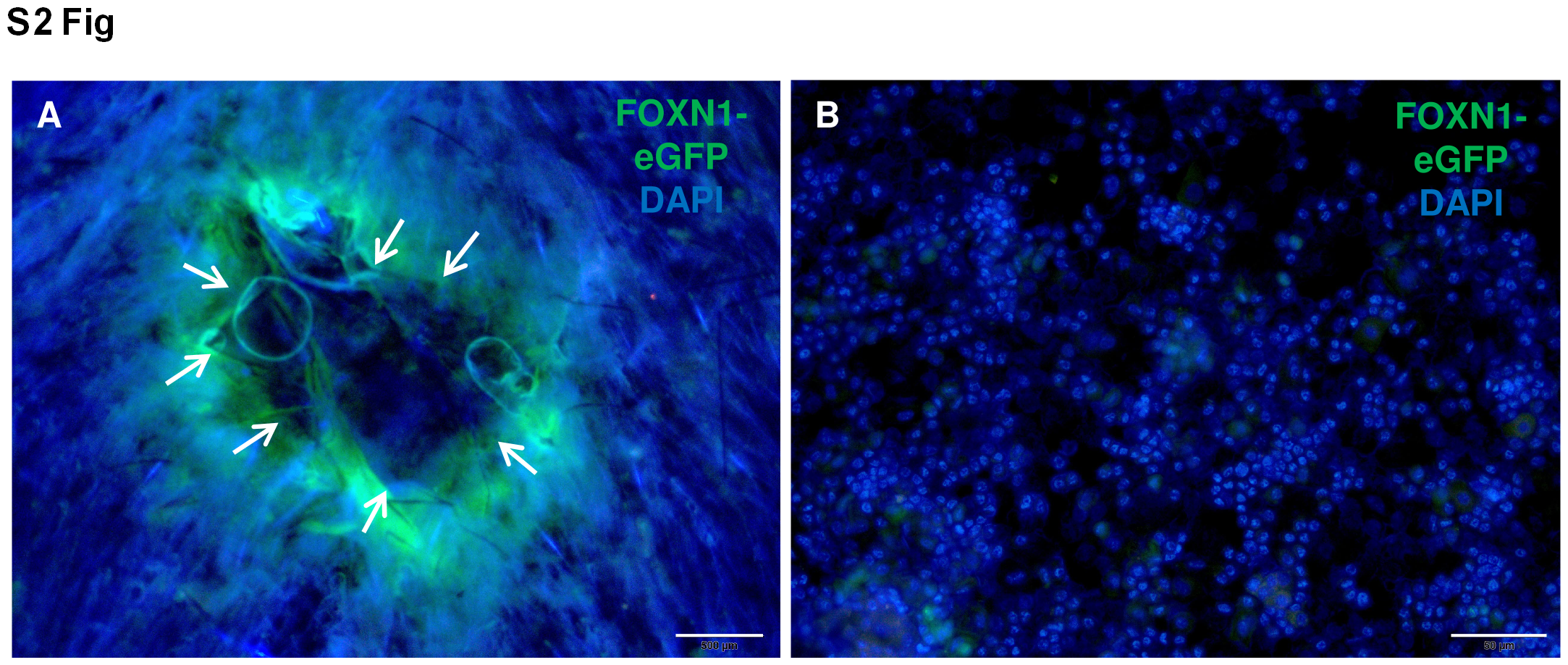

Supplement: S2 Fig — Whole mounts dorsal epidermis from Foxn1::Egfp mice of postinjured (A) and uninjured (B) skin area at day 5 post-injury. Arrows indicate wound margin. Scale bar 500 μm (A) and 50 μm (B). (TIF) [file pone.0150635.s002.tif]

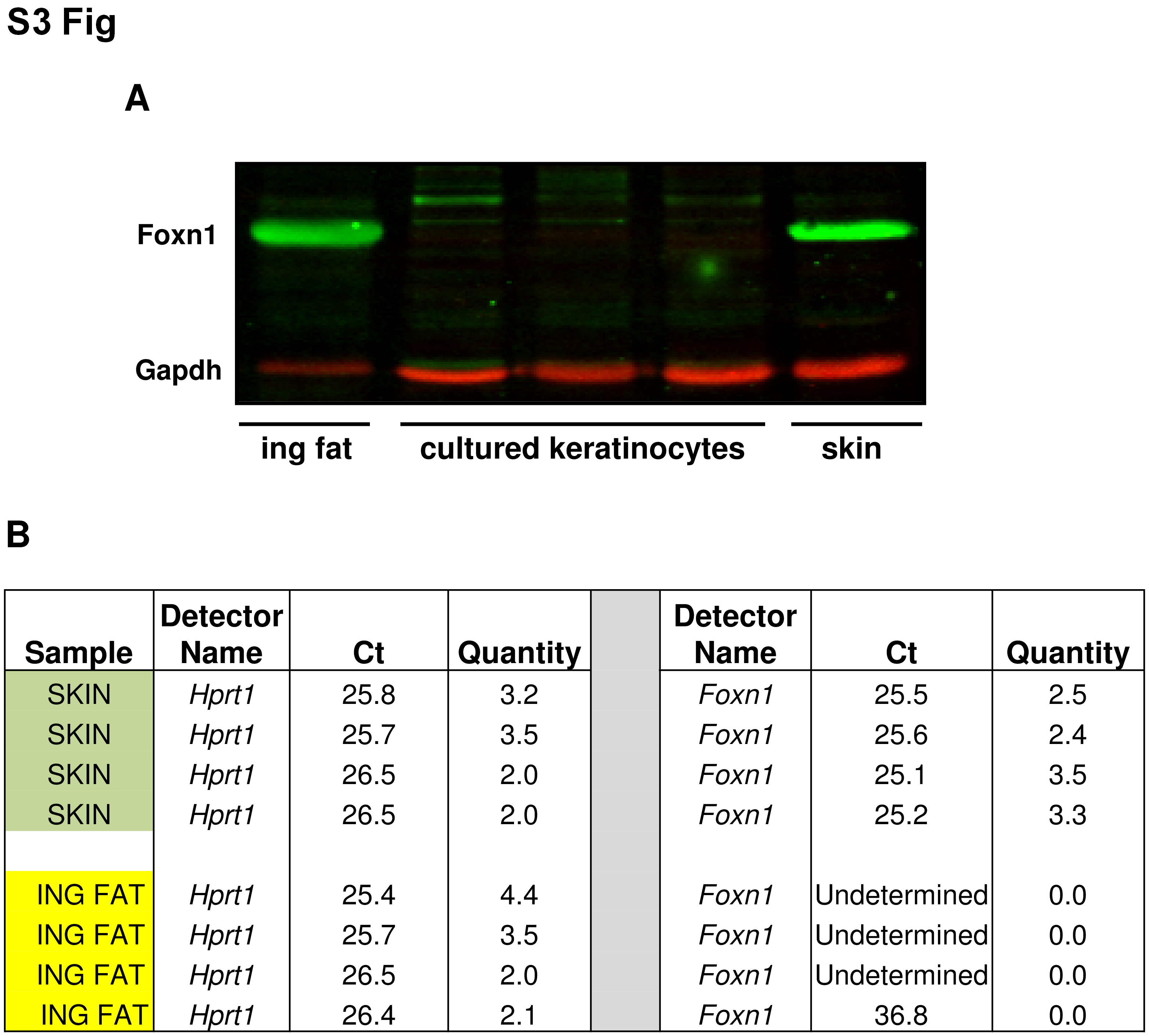

Supplement: S3 Fig — Detection of Foxn1 protein with Western Blot (A) and Foxn1 mRNA with qRT-PCR (B) analysis. False positive Foxn1 protein band present in inguinal fat and skin tissues (A). Expression of Hprt1 mRNA in skin and inguinal fat tissues, and Foxn1 mRNA in skin tissues (B). (TIF) [file pone.0150635.s003.tif]
