# Supplementary material for: Foxn1 Transcription Factor Regulates Wound Healing of Skin through Promoting Epithelial-Mesenchymal Transition
Source: PLoS One. 2016 Mar 3;11(3):e0150635. doi: 10.1371/journal.pone.0150635 (PMC4777299; doi:10.1371/journal.pone.0150635)
Supplement: S1 Protocol — Foxn1::Egfp (n = 3) and B6 (n = 1; negative immunofluorescent control) animals were used for whole mount slide analysis. 8 mm diameter post-injured and uninjured skin tissues were collected, washed in PBS, incubated overnight in 3.33 mg/ml dispase enzyme (1.89U/mg) (Life Technologies) in Hanks' balanced salt solution (HBSS; Sigma) at 4°C. Then, skin samples were placed in pre-warmed (37°C) 0.05% trypsin-EDTA solution (Trypsin-EDTA 1x, Life Technologies) for 10 min. Epidermis were separated from the dermis as an intact sheet and was placed on glass slides fixed in 4% PFA for 20 min, washed in PBS and mounted in ProLong® Gold Antifade Mountant with DAPI (Life Technologies). Sections were visualized with an Olympus microscope (BX43) and photographed with an Olympus digital camera (XC50). (DOCX) [file pone.0150635.s004.docx]

**S2 Supplementary Material.** Epidermal whole-mounts protocol.

Foxn1::Egfp (n=3) and B6 (n=1; negative immunofluorescent control) animals were used for whole mount slide analysis. 8 mm diameter post-injured and uninjured skin tissues were collected, washed in PBS, incubated overnight in 3.33 mg/ml dispase enzyme (1.89U/mg) (Life Technologies) in Hanks' balanced salt solution (HBSS; Sigma) at 4°C. Then, skin samples were placed in pre-warmed (37°C) 0.05% trypsin-EDTA solution (Trypsin-EDTA 1x, Life Technologies) for 10 min. Epidermis were separated from the dermis as an intact sheet and was placed on glass slides fixed in 4% PFA for 20 min, washed in PBS and mounted in ProLong® Gold Antifade Mountant with DAPI (Life Technologies). Sections were visualized with an Olympus microscope (BX43) and photographed with an Olympus digital camera (XC50).
